# Blood biomarkers and artificial intelligence: toward precision medicine in Alzheimer’s disease

**DOI:** 10.3389/fnagi.2026.1937244

**Published:** 2026-09-23

**Authors:** Linhong Xu, Jianbo Tu, Jipeng Zhang, Chen Zhang, Yongan Sun

**Affiliations:** 1 Department of Neurology, Peking University First Hospital, Peking University, Beijing, China; 2 Health Service Department of the Guard Bureau of the Joint Staff Department, Beijing, China; 3 Health Service Department of the Guard Bureau of the General Office of the Central Committee of the Communist Party of China, Beijing, China

**Keywords:** Alzheimer’s disease, artificial intelligence, blood-based biomarkers, machine learning, precision diagnosis

## Abstract

The integration of blood-based biomarkers and artificial intelligence (AI) is revolutionizing the diagnostic landscape of Alzheimer’s disease (AD). In this paper, we synthesize recent advancements in the application of machine learning (ML) on blood biomarker analysis, evaluating their diagnostic efficacy and clinical translational potential. Current evidence identifies plasma p-tau217 as the best individual biomarker for primary screening. However, multiple biomarker signatures (such as A/T/N) combined with ML algorithms (like XGBoost) greatly improve diagnostic accuracy for mild cognitive impairment. Notably, predictive performance is maximized with multimodal fusion, which integrates data from blood, neuroimaging, and genetics, achieving an area under the curve of up to 0.94. To mitigate the black-box nature of these models, explainable AI frameworks like SHapley Additive exPlanations (SHAP) are essential for establishing clinical interpretability. Ultimately, we propose a scalable “Step-Care” framework that transitions from single-biomarker community screening to precise multimodal fusion, optimizing clinical trial stratification and personalized workflows.

## 1 Introduction

Alzheimer’s disease (AD) is the most common neurodegenerative disorder. With the rapid aging of the global population, AD has become a serious threat to public health (Liu et al., 2024b). By 2050, the global prevalence of AD could reach 152.8 million (Nichols et al., 2022). Alzheimer’s disease manifests as chronic progressive cognitive impairment, imposing a severe economic burden on society (Gläser et al., 2025). Conventional diagnostic methods for Alzheimer’s Disease typically include neuropsychological assessment, neuroimaging assessment, and cerebrospinal fluid analysis (Self and Holtzman, 2023). However, their time-consuming nature, high cost, and invasiveness limit extensive testing.

Recently, blood biomarkers have emerged as an innovative, minimally invasive method for detecting AD. For instance, phosphorylated tau 181 (p-tau181), p-tau217, and p-tau231 show high accuracy in distinguishing AD pathology; specifically, p-tau217 correlates more strongly with amyloid beta plaques and tau protein tangles (Hansson et al., 2023). The plasma Aβ42/Aβ40 ratio shows a decrease in AD patients and exhibits alterations even in individuals with mild cognitive impairment (MCI) (Pérez-Grijalba et al., 2019; Chatterjee et al., 2023). Additional blood-based biomarkers, including neurofilament light chain (NfL) and glial fibrillary acidic protein (GFAP), provide insights into disease progression (Prins et al., 2022; Wojdała et al., 2023). However, blood biomarkers are still challenged by low concentrations, pre-analytical variability, lack of assay standardization, and the confounding impact of systemic comorbidities (Schöll et al., 2024).

Recently, artificial intelligence (AI) and its subset, machine learning (ML), have made a great impact on computational medicine. For instance, due to their ability to parse high-dimensional, non-linear relationships within large amounts of data, ML models are used to optimize diagnostics of Alzheimer’s disease, refine clinical trial design, and accelerate drug discovery (Geng et al., 2024). Unlike traditional statistics, deep learning (DL) can discover hidden patterns in massive and complex datasets (Esteva et al., 2019), thereby enhancing diagnosis and early detection (Qiu et al., 2022). The combination of AI/ML and blood biomarkers facilitates the creation of robust early-warning diagnostic systems.

By connecting advanced computational tools with real-world clinical practice, this study reviews recent advances in integrating blood biomarkers and AI for AD, offering insights into personalized clinical practice.

## 2 Materials and methods

### 2.1 Literature search strategy

A comprehensive literature search was conducted across PubMed, Embase, and Web of Science. The search terms included: ((“Alzheimer*” OR “cognit*”) AND ((“blood”[TIAB] OR “plasma”[TIAB] OR “serum”[TIAB]) AND (“biomarker*”[TIAB] OR “marker*”[TIAB])) AND (“Machine Learning”[Mesh] OR “Artificial Intelligence”[Mesh])) AND (“tau” OR “p-tau” OR “t-tau” OR “Aβ42” OR “Aβ40” OR “Aβ” OR “βA” OR “amyloid” OR “β amyloid” OR “β-amyloid” OR “amyloid-β” OR “amyloid β” OR “NFL” OR “neurofilament light chain” OR “GFAP” OR “glial fibrillary acidic protein”) NOT (“animals”[MH] NOT “humans”[MH]). We focused on literature published between January 2019 and 8th September 2025 to capture the most recent advances in this rapidly evolving field. Additionally, a supplementary search was performed by examining the reference lists of key review articles to identify seminal publications. This review was conducted in accordance with the Preferred Reporting Items for Systematic Reviews and Meta-Analyses (PRISMA) guidelines (Page et al., 2021). The PRISMA flow diagram (Supplementary Figure 1) and the checklist (Supplementary Table 1) are provided in the Supplementary material.

### 2.2 Literature selection criteria

The selection of literature was guided by the following principles to identify studies that are representative and impactful to the field, rather than solely on rigid systematic criteria.

Topic Relevance: Priority was given to original research that directly focused on applying ML models to blood biomarker data for AD diagnosis, classification, or risk prediction. The blood-based biomarkers included, but were not limited to, amyloid β-protein (Aβ), p-tau, NfL, and GFAP. Human participants clinically diagnosed with Alzheimer’s disease, mild cognitive impairment, subjective cognitive decline, healthy control groups, or patients with other neurodegenerative diseases were included. Datasets from community-based or clinical cohorts were included. This review focuses specifically on machine learning modeling approaches only, which include conventional ML modeling, deep learning modeling, ensemble and multimodal modeling, as well as feature selection and model interpretability modeling approaches.

Study design and quality: High-quality peer-reviewed cross-sectional and prospective cohort studies were included. Selected review articles and authoritative guidelines were also included to help establish the background context.

Exclusion criteria primarily consisted of non-English/Chinese publications, conference abstracts lacking complete data, non-peer-reviewed preprints, animal studies, interventional research, studies using neuroimaging or cerebrospinal fluid (CSF) data without integrating blood biomarkers, and studies lacking quantifiable model performance metrics.

### 2.3 Data extraction and synthesis

All records from the database searches were exported to Zotero for de-duplication. The selection procedure was conducted by two reviewers through a title/abstract review and a subsequent full-text review of potentially relevant articles. Data were extracted using a structured data extraction form with items for author, year, data sources, cohort name, target population, sample size, plasma biomarkers, analysis techniques, optimal diagnostic model(s) and their performance, and conclusions. A qualitative narrative synthesis was performed; we categorized extracted data to compare diagnostic strategies from single-biomarker to multimodal fusion.

We used the modified Quality Assessment of Diagnostic Accuracy Studies-2 (QUADAS-2) tool to assess the risk of bias (Whiting et al., 2011; Wade et al., 2013). The following four domains were evaluated: (1) patient selection, (2) biomarker measurement, (3) reference standard, and (4) flow and timing. The study was rated as low, unclear, or high risk of bias. Because several included studies involved prognostic prediction and machine learning models rather than conventional diagnostic accuracy designs, relevant considerations from PROBAST were also taken into account (Wolff et al., 2019).

## 3 Results

A total of 447 records were identified from PubMed (*n* = 50), Embase (*n* = 218), and Web of Science (*n* = 179). After removing 131 duplicates, 316 records were screened based on titles and abstracts. Of these, 269 records were excluded as not relevant to the topic, non-original studies, or not in English/Chinese. The remaining 47 full-text articles were assessed for eligibility, of which 24 were excluded after full-text review, including animal studies, interventional studies, studies using only neuroimaging or CSF data without integrating blood biomarkers, studies lacking quantifiable model performance metrics, and other studies not meeting inclusion criteria. Ultimately, 23 studies met the inclusion criteria. The detailed selection process is summarized in Supplementary Figure 1. Detailed results of risk of bias assessment are shown in Supplementary Figures 2, 3. The characteristics of the included studies are summarized in Table 1.

**TABLE 1:** Characteristics of included studies.

References	Study cohort	Population	Sample size	Plasma biomarkers	Analyzing methods	Best diagnostic model	Conclusion
Gao et al., 2023	CANDI	CU, MCI, AD, non-AD dementia.	411	Aβ40, Aβ42, p-tau181, t-tau.	LASSO regression, gradual regression.	Plasma p-tau + APOE genotype + MRI index. AUC = 0.914.	The model can replace expensive/invasive tests for early screening of Alzheimer’s disease.
Park et al., 2024	ADNI	CU and MCI.	349	Whole blood RNA sequencing, p-tau181, NFL.	Ridge regression, LASSO regression, SVM, RF. 50% off cross-validation.	CU vs MCI: SVM (transcription data only). AUC = 0.98. MCI vs AD: Ridge regression. AUC = 0.96.	Early Diagnosis: The SVM model differentiated MCI from CU. 123 RNA markers along with p-tau181/NFL can identify high-risk MCI patients.
Ngai et al., 2024	UKB	AD and controls.	502,364	GFAP, CXCL17, etc. 249 metabolites.	LGBM, CatBoost, XGB. DL: TabNet. Baseline Models: LR, linear regression. Multi-modal Fusion Strategies: Early Fusion and Late Fusion.	Case-Control: Early fusion CatBoost. Performance: AUROC = 0.87, AUPRC = 0.62. AAO: Late fusion LGBM. MSE = 43.28.	Multi-omics fusion models effectively predicted AD risk and AAO, outperforming existing genetic risk assessments.
Wang et al., 2025	ADNI	CU and MCI.	239	APOE, CRP, IL16, MMP1, etc.	Traditional models: SVM, LR, NB, RF, KNN, GBM, and XGB. Feature Selection: LASSO algorithm. DL: DNN.	DL Model: Activation function: “Rectifier With Dropout”; 2 hidden layers; 32 input features; Accuracy = 0.995; F1-score = 0.996.	DNN outperformed traditional ML methods in MCI prediction, and the combination of plasma protein biomarkers achieved high accuracy for early diagnosis.
Jiao et al., 2025	Xiangya Hospital	AD, MCI, DLB, FTD, PSP and HC.	1324	p-tau217, Aβ42, GFAP. Spectral Biomarkers.	Feature Selection: Relief-F. Classification Model: RF, SVM, KNN, etc. Sample Partitioning: Kennard-Stone.	6 AD spectra digital biomarkers for Aβ42, p-tau, and GFAP. AD vs. HC: AUC = 0.92. MCI vs. HC: AUC = 0.89.	This study is the first to combine ATR-FTIR spectroscopy with machine learning to develop a digital biomarker panel for AD, supporting early diagnosis in resource-limited settings.
Eke et al., 2021	ADNI	AD, MCI and HC.	356	146 plasma proteins: A2M, APOE, BNP, etc.	SVM. Feature Selection: CFS. Brute-Force Search. Model Evaluation: 10-fold cross-validation repeated 10 times.	A2M + APOE + BNP + Eotaxin-3 + RAGE + SGOT. Quadratic polynomial kernel SVM. MCI vs. HC: AUC ≥ 0.80. AD vs. HC: AUC = 0.90–0.94.	Confirmed the feasibility of using non-amyloid blood biomarker panels for early AD detection, enabling risk identification before amyloid plaque formation.
Karlsson et al., 2025	Swedish BF2, Swedish BF1, ADNI, UCSF-ADRC, A4, OASIS-3	CU, SCD, MCI, AD Dementia.	1797.	p-tau217, p-tau181, p-tau231, NTA, GFAP, NfL (Simoa).	CatBoost, RF, support vector regression SVR, XGB. Feature selection: RFE, RF, etc. Validation methods: 10-fold cross-validation.	Tau-PET Load Prediction: CatBoost regressor, R^2^= 0.66–0.69, MAE = 0.16–0.17 SUVR. Tau-PET Asymmetry Prediction: SVR, R^2^= 0.28–0.42, MAE = 6.7–9.6.	Blood biomarkers can efficiently predict tau-PET load. MRI structural data can capture features of tau spatial distribution but may underestimate tau load when used alone.
Chiu et al., 2022	Taiwan University Hospital	SCD, MCI and AD.	206	Aβ42, Aβ40, tau.	Feature extraction: LDA. Feature selection: SFS. Classification algorithms: RF and SVM, evaluated using LOOCV.	12-feature model: MMSE total score, age, plasma Aβ40, etc. RF model: AUROC = 0.86–0.90. SVM model: Corresponding AUROC = 0.80–0.90.	Combining plasma biomarkers with brain MRI structural data through machine learning can distinguish early stages of AD, providing a non-invasive auxiliary tool for clinical diagnosis.
Deivasigamani et al., 2025	FCA3DS, IADRC, Public datasets ANMerge	CU and AD.	323	7,298 plasma proteins, p-tau181.	Nested cross-validation with elastic net, RF, etc. Feature selection: t-test, RF variable importance, PLS, and Relief-F algorithm. WGCNA.	36-protein predictive model: AUC = 0.94 in distinguishing AD from CU.	Untargeted proteomics and machine learning study identified a 36-protein panel that distinguishes AD, validated across multiple cohorts.
Chang et al., 2025	Kaohsiung Chang Gung Memorial Hospital, Taiwan	CU, AD and non-AD neurodegenerative disease patients.	371	p-tau217, p-tau181, GFAP, NFL, Aβ42/40 ratio, total tau.	Multiclass LR, SVM, RF. Statistical Methods: ROC curve analysis, AUC comparison (DeLong test), feature importance ranking. Cross-Validation: 5-fold cross-validation and random splitting.	Multiclass LR and RF: Accuracy>92% for identifying A+, reaching 93.4% in early stages. P-tau217 Single: AUC = 0.947, accuracy > 90%.	Single p-tau217 has accuracy comparable to combined panels. Combining biomarkers with machine learning models is recommended for early AD detection and patient stratification.
Vergallo et al., 2019	INSIGHT-preAD study, part of APMI-CP	Cognitively normal but had persistent SMC.	276	Aβ40, Aβ42, Aβ40/Aβ42,t-Tau, NFL, YKL-40, BACE1.	Hypothesis-Driven Analysis: ROC curves and DeLong test. Hypothesis-Free Machine Learning: RF. CART.	CART Model: Plasma Aβ40/Aβ42 ratio. Training set: Balanced accuracy 81%. Test set: Balanced accuracy 71%.	Plasma Aβ40/Aβ42 ratio serves as a low-cost, non-invasive screening tool for cerebral amyloid deposition, suitable for early stratification of at-risk AD populations.
Liu et al., 2024a	ADNI	CU, MCI and AD.	803	Plasma microRNAs.	STREAMLINE automated pipeline (RF, LR, etc.). Feature importance evaluation: Mutual information and MultiSURF. Performance metric: ROC-AUC (5-fold cross-validation).	RF: mean ROC-AUC: Group A 0.68, Group T 0.62, Group N 0.64. Performance Improvement: Addition of miRNAs improved classification performance by 9% (from 0.59 to 0.68).	Plasma miRNAs have potential as non-invasive blood biomarkers. miRNAs can improve AD pathological classification performance, providing new insights for early diagnosis.
Huang et al., 2025	IADRC, FCA3DS, ADNI	CU, MCI and AD.	498	Aβ42/40, p-tau181, NfL, GFAP. 7,181 plasma protein aptamers.	STREAMLINE pipeline: elastic net, RF, etc. Linear regression; Protein Co-expression Network (WGCNA); Pathway Enrichment (Metascape).	Model 3 (Elastic Net Algorithm): Demographics + A/T/N/I + 20 proteins (e.g., NPTXR). AUC = 0.930.	Plasma protein abnormalities reflect AD molecular mechanisms. Differentially expressed proteins improve AD diagnostic performance.
Mohammed and Malhotra, 2025	ADNI, OASIS, specialized hospitals in India	CU, MCI, and AD.	3100	p-tau181, Aβ42, NfL, YKL-40.	Multimodal fusion framework: Integrates CNN, LSTM, and GNN. ComBat correction, CycleGAN. Nested cross-validation (5-fold).	Multimodal model (integrating MRI, PET, plasma biomarkers, etc.): AUC-ROC = 0.94 (ADNI), 0.93 (OASIS).	The multimodal ML framework improves early AD diagnostic accuracy, particularly relying on hippocampal volume and plasma p-tau.
Chen et al., 2025	Guangzhou Health Aging and Dementia Cohort	CU, MCI and AD.	349	Aβ40, Aβ42, p-tau181, GFAP, NfL.	Logistic and linear regression models. Five-fold cross-validation. Statistical Tools: R 4.2.2 and MATLAB.	Linear regression. MemTrax+p-tau181/Aβ42: predicting CL values. MemTrax + p-tau181: AUC = 0.880.	MemTrax combined with blood biomarkers enables non-invasive, low-cost, and accurate prediction of Aβ burden.
Shah et al., 2025	Mayo Clinic Study of Aging	CU, MCI and Dementia.	1254	Aβ42/Aβ40 ratio, GFAP, NfL, p-tau181.	LR, RF, XGB, etc. with parameters optimized via 5-fold cross-validation and grid search. Stepwise feature addition strategy.	Plasma + NPS: Best predicted visuospatial skills decline.	Machine learning models combining NPS with AD biomarkers slightly improved prediction of cognitive decline, though effect sizes were modest.
Youn et al., 2022	ADAM cohort at SNU Bundang Hospital in South Korea	SCD, MCI, AD, non-AD dementia.	312	Amyloid-β oligomerization tendency based on EDTA plasma.	SVM, RF, LR, DNN. Evaluation Metrics: Accuracy, Precision, Recall, F1-score, with 50 repetitions of cross-validation.	RF Model, MDS-OAβ + MMSE + APOE + Age. Performance: Accuracy = 77.14 ± 4.21%, F1-score = 85.44 ± 3.10%.	MDS-OAβ in EDTA plasma, combined with machine learning, can serve as a screening tool for amyloid PET positivity, reducing the need for invasive testing.
Vintimilla et al., 2025	HABS-HD cohort at UNTHSC, USA	CU.	952	Aβ40, Aβ42, Aβ42/40, total Tau, NFL, CRP, ICAM, VCAM.	RF regression models. Gini importance. RMSE and R^2^. Multiple imputation using PMM.	The RF model performed excellently across all cognitive domains. R^2^≈0.94–0.98, RMSE ≤ 0.5.	Cognitive performance is influenced by social, economic, biological, and functional factors.
Kim et al., 2024	PNU Hospital and CNU Hospital	AD and HC.	40	Aβ42 and its oligomers. Metabolites: NeuAc, uric acid, etc.	DL: Three-layer feedforward neural network, ReLU activation, cross-entropy loss, stochastic gradient descent optimizer. IG.	Amino-SAM Substrate + Deep Learning: Accuracy = 99.5%.	The amino-SAM-modified substrate combined with deep learning effectively differentiated AD from HC, offering a non-invasive approach for early AD diagnosis.
Zhao et al., 2024	ADNI, SHAPE	CU and MCI.	111	GFAP, NFL, GFAP/NFL.	Feature selection: LASSO regression. Classification models: XGB, RF, Decision Tree Classifier. Validation: Five-fold stratified cross-validation.	Left/right amygdala SUVR, left entorhinal cortex SUVR, age, years of education, plasma NfL, plasma GFAP, GFAP/NfL ratio. RF. AUC = 0.887.	Plasma biomarkers and tau-PET imaging effectively distinguish MCI. Tau in the amygdala and entorhinal cortex is a key mediator through which biomarkers affect cognitive function.
Galvin et al., 2025	Bio-Hermes study (NCT04733989)	CU, MCI, AD, non-AD dementia.	887	Aβ40, Aβ42, Aβ40/Aβ42, p-tau181, p-tau217.	GBM regression model, Support Vector Machine, and Random Forest.	CARM Score (4-point scale): CARM1: Specificity 80%. CARM3/4: Sensitivity 80%.	Cognivue Clarity combined with CARM enables low-cost screening for cognitive impairment and amyloid risk, optimizing early AD intervention and clinical trial enrollment.
Duggan et al., 2025	ARIC, NILS-LSA, BLSA	All participants were dementia-free at baseline.	12831	≈5,000 plasma proteins. Aβ40/Aβ42, GFAP, NfL, p-tau181.	Cox proportional hazards models. LASSO regression. AFT model to generate a continuous risk score (dSST).	dSST Model: 25-protein panel; AUC = 0.68–0.70 (20-year risk), 0.78 (5-year risk).	A low-cost, scalable plasma proteomic prediction tool (dSST) was developed, capable of quantifying dementia risk up to 20 years before symptom onset.
Shima et al., 2025	Fujita Health University Aging Registry Study (Japan)	All participants were cognitively normal.	111	GFAP, NfL, Aβ40, Aβ42, p-tau181.	LR, linear kernel SVM, Gaussian kernel SVM, RF. LOOCV. MCR. Univariate analysis. Multivariate linear/ Logistic regression.	Linear Kernel SVM: Accuracy = 74%, AUC = 0.73. ROC Analysis: p-tau181 ≥ 2.2 pg/mL predicted PI error; AUC = 0.86.	PI error measured by the 3D VR navigation system, especially when combined with plasma p-tau181, can serve as a non-invasive marker for early AD pathology.

A2M, Alpha-2-Macroglobulin; A4, The Anti-Amyloid Treatment in Asymptomatic Alzheimer’s Disease Study; AAO, Age at Onset; AD, Alzheimer’s disease; ADNI, Alzheimer’s Disease Neuroimaging Initiative; AFT, accelerated failure time; APMI-CP, the Alzheimer Precision Medicine Initiative–Core Protocol; APOE, Apolipoprotein E; ARIC, Atherosclerosis Risk in Communities; ATR-FTIR, Attenuated Total Reflectance - Fourier Transform Infrared Spectroscopy; AUC, Area Under the Curve; AUPRC, Area Under the Precision-Recall Curve; AUROC, Area Under the Receiver Operating Characteristic curve; BF, BioFINDER; BLSA, Baltimore Longitudinal Study of Aging, U.S; BNP, B-type Natriuretic Peptide; CANDI, China Aging and Neurodegenerative Initiative; CARM, Cognivue Amyloid Risk Measure; CART, Classification and Regression Tree; CatBoost, Categorical Boosting; CFS, Correlation-Based Feature Selection; CgA, Chromogranin A; CNN, Convolutional Neural Network; CNU, Chungnam National University; CRP, C-Reactive Protein; CSF, Cerebrospinal Fluid; CU, Cognitively Unimpaired; CXCL, C-X-C Motif Chemokine Ligand; DL, Deep Learning; DLB, Dementia with Lewy Bodies; DNN, deep neural network; dSST, digit Symbol Substitution Test; EDTA, Ethylenediaminetetraacetic Acid; FCA3DS, Florida Consortium of African American Alzheimer’s Disease Study; FTD, Frontotemporal Dementia; GFAP, glial fibrillary acidic protein; GNN, graph neural networks; HABS-HD, Health and Aging Brain Study, Health Disparities; HC, healthy control; IADRC, Indiana Alzheimer’s Disease Research Center; ICAM, Intercellular Adhesion Molecule; IG, Integrated Gradients; IGF-1, Insulin-like Growth Factor 1; KNN, K-Nearest Neighbors; LASSO, Least Absolute Shrinkage and Selection Operator; LDA, Linear discriminant analysis; LGBM, Light Gradient Boosting Machine; LOOCV, leave-one-out cross-validation; LR, Logistic Regression; LSTM, Long Short-Term Memory; MAE, Mean Absolute Error; MCI, mild cognitive impairment; MCR, Model Class Reliance; MDS-OAβ, Movement Disorder Society - Outcome Assessment in Offspring of Autosomal Dominant Alzheimer’s Disease Trials; ML, Machine Learning; MMP, Matrix Metalloproteinase; MMSE, Mini-Mental State Examination; MoCA, Montreal Cognitive Assessment; MRI, magnetic resonance imaging; NB, Naïve Bayes; NfL, neurofilament light chain; NILS-LSA, National Institute for Longevity Sciences - Longitudinal Study of Aging; NPS, Neuropsychiatric Inventory; NPTXR, Neuronal Pentraxin Receptor; OASIS-3, Open Access Series of Imaging Studies-3; PET, Positron Emission Tomography; PHS, Pittsburgh Sleep Quality Index; PI, path integration; PLS, partial least squares; PNU, Pusan National University; PSP, Progressive Supranuclear Palsy; RAGE, Receptor for Advanced Glycation End products; ReLU, Rectified Linear Unit; RF, Random Forest; RFE, Recursive Feature Elimination; RMSE, Root Mean Square Error; SAM, Self-Assembled Monolayer; SCD, Subjective cognitive complaints but normal cognitive test results; SFS, Sequential forward selection; SGOT, Serum Glutamic-Oxaloacetic Transaminase; SHAPE, Shanghai Action of Prevention Dementia for the Elderly; SMC, subjective memory complaints; SUVR, Standardized Uptake Value Ratio; SVM, Support Vector Machine; SVR, Support Vector Regression; TabNet, Tabular Network; UCSF-ADRC, University of California, San Francisco - Alzheimer’s Disease Research Center; UKB, UK Biobank; UNTHSC, the University of North Texas Health Science Center; VCAM, Vascular Cell Adhesion Molecule; VEGFR, Vascular Endothelial Growth Factor Receptor; WGCNA, Weighted gene co-expression network analysis; XGB, eXtreme Gradient Boosting.

### 3.1 Blood-based biomarkers

#### 3.1.1 Single biomarkers

Phosphorylated Tau (p-tau) proteins (specifically p-tau217) have demonstrated remarkable accuracy in AD diagnosis. Among the literature reviewed, p-tau181 and p-tau217 have been applied in diagnostic or early staging models for AD. A multicenter cross-sectional study (*n* = 1797) revealed that plasma p-tau217 was the strongest predictor of brain tau protein deposition load, outperforming conventional clinical and magnetic resonance imaging (MRI) variables (Karlsson et al., 2025). A prospective cohort study (*n* = 371) further confirmed that p-tau217 alone achieved a performance comparable to multi-marker panels, highlighting its suitability as a front-line screening tool (Chang et al., 2025).

The Aβ42/Aβ40 ratio remains critical for detecting early amyloid pathology, yielding an area under the curve (AUC) of 0.794 (*n* = 276) (Vergallo et al., 2019). Conversely, individual Aβ40 and Aβ42 demonstrate poor diagnostic sensitivity (Vergallo et al., 2019). GFAP, a key indicator of astrocyte activation, correlates positively with medial temporal lobe tau deposition (Zhao et al., 2024). Additionally, NfL functions as an indicator of neuroaxonal damage and effectively predicts cognitive decline and disease progression (Park et al., 2024).

Advanced bioanalytical platforms have further identified novel biomarkers. For instance, a novel plasma assay measuring amyloid-beta oligomers showed an accuracy of 71.09% in predicting amyloid positron emission tomography (PET) positivity (Youn et al., 2022). Furthermore, proteomic screens have identified synaptic proteins that are dysregulated, such as NPTXR and CPLX2, as highly sensitive indicators for early synaptic dysfunction in AD (Deivasigamani et al., 2025), while metabolomics identified specific alterations in N-acetylneuraminic acid in AD (Kim et al., 2024).

#### 3.1.2 Biomarker panels

The combination of various biomarkers that reflect different pathophysiological processes yields synergistic effects. Aligning with the established biological A/T/N (Amyloid/Tau/Neurodegeneration) framework, the combination of p-tau217, p-tau181, GFAP, NfL, Aβ42/Aβ40 ratio, and total Tau showed an outstanding accuracy of 93.4% (Chang et al., 2025). Similarly, an expanded A/T/N/I (incorporating inflammation) panel achieved an AUC above 0.90 in diverse diagnostic classification tasks (Huang et al., 2025).

High-throughput discovery proteomics studies have shown increased accuracy capacity. A validated panel of 36 proteins differentiated AD from cognitively normal controls with an AUC of 0.94 (Deivasigamani et al., 2025). For long-term risk prediction, a 25-protein panel has shown great potential in the prediction of dementia onset up to 20 years before symptomatic manifestation (Duggan et al., 2025). Furthermore, an optimized 6-protein panel achieved a sensitivity > 80% and specificity > 70% for early-stage detection (Eke et al., 2021).

#### 3.1.3 Multimodal models

The combination of blood biomarkers, genetic information, and neuroimaging proves to be exceptionally promising. An AUC of 0.914 has been obtained in predicting amyloid PET positivity using plasma p-tau, apolipoprotein E (APOE) status, and MRI metrics (Gao et al., 2023). Similarly, blood biomarkers of Aβ and tau, together with the use of structural MRI, can successfully map individuals across the continuous AD spectrum (Chiu et al., 2022).

Moreover, blood biomarkers used in conjunction with digital cognitive tests offer an efficient option for routine use. For instance, a 2-min online memory test (MemTrax) combined with the p-tau181/Aβ42 ratio yielded an AUC of 0.880 for predicting brain amyloid burden (Chen et al., 2025). Similarly, using a Gradient Boosting Machine integrated cognitive test scores and age yielded an effective Cognivue Amyloid Risk Measure (CARM) for risk stratification (Galvin et al., 2025). Lastly, incorporating the neuropsychiatric inventory into plasma markers enhanced the prediction of cognitive decline, particularly within language and visuospatial dimensions (Shah et al., 2025).

### 3.2 Artificial intelligence and algorithmic analysis

The use of blood biomarkers and artificial intelligence/machine learning in Alzheimer’s disease is summarized in Figure 1. Briefly, the left column (Plasma Biomarkers) categorizes biomarkers into four groups: Aβ pathology-related markers, tau pathology-related markers, neurodegeneration and inflammation markers, and novel multi-omics biomarkers. The middle column (Artificial Intelligence Analysis) encompasses four analytical approaches: traditional ML models and feature selection methods; deep learning architectures; multi-modal fusion strategies; and explainable AI techniques. The right column (Applications) outlines four key clinical applications: early screening and stratification in community/primary care settings; disease progression prediction; precision medicine and clinical trial stratification; and low-resource setting adaptation.

**FIGURE 1:**
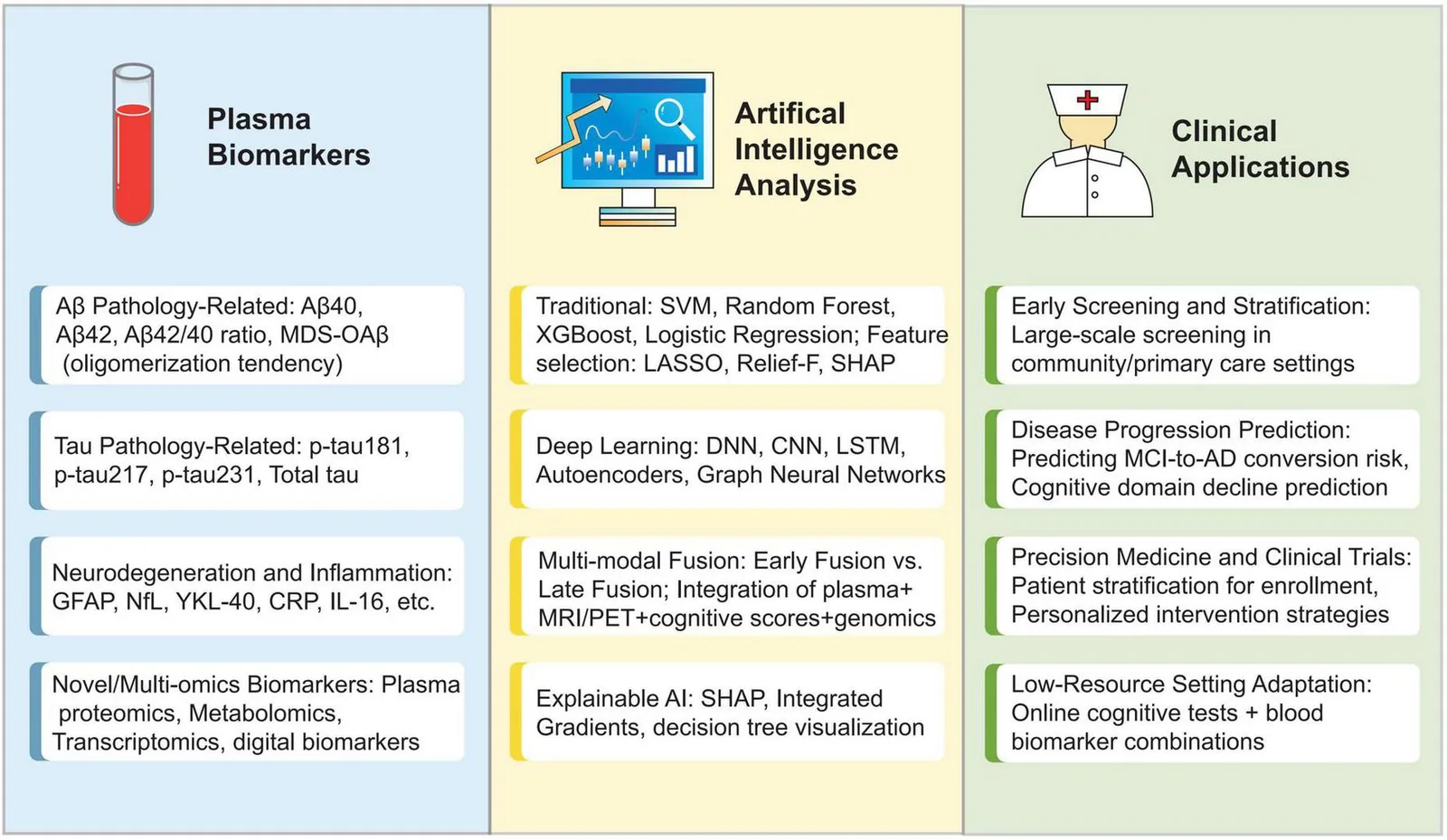
The application of blood-based biomarkers and artificial intelligence in the diagnosis and staging of Alzheimer’s disease. The diagram illustrates the integration of three core modules, from data acquisition to clinical translation. AD, Alzheimer’s disease; Autoencoders, unsupervised neural networks for dimensionality reduction; CNN, Convolutional Neural Network; CRP, C-reactive protein; DNN, deep neural network; GFAP, glial fibrillary acidic protein; IL-16, Interleukin-16; LASSO, Least Absolute Shrinkage and Selection Operator; LSTM, Long Short-Term Memory; MCI, mild cognitive impairment; MDS-OAβ, Movement Disorder Society-Oligomeric Amyloid-beta; NfL, neurofilament light chain; Relief-F, Relief Feature selection; SHAP, SHapley Additive exPlanations; SVM, Support Vector Machine; YKL-40, Chitinase-3-like protein 1.

#### 3.2.1 Traditional machine learning models

Tree-based ensemble models often show superior performance in classification analysis. XGBoost showed extraordinary accuracy for discriminating between clinical normal (CN) and MCI cases with accuracies and F1-scores of 0.986 and 0.985, respectively, by using 35 plasma proteins (Wang et al., 2025). Similarly, the CatBoost regressor proved optimal for quantifying tau-PET burden, with plasma p-tau217 being the most relevant variable (Karlsson et al., 2025).

Support Vector Machines (SVMs) exhibit distinct advantages in processing high-dimensional data. Utilizing blood transcriptomics alone, an SVM reached an AUC score of 0.98 for distinguishing between Cognitively Unimpaired and MCI patients (Park et al., 2024). Furthermore, an SVM with a quadratic polynomial kernel using a 6-protein panel achieved sensitivities above 80% and specificities above 70% for discriminating MCI from Healthy controls (Eke et al., 2021).

Regularized linear models are useful for feature selection and parsimony. In multi-modal tasks combining transcriptomic, demographic, and neuropsychological information to classify MCI from AD, Ridge Regression emerged with extraordinary performance (AUC = 0.96) (Park et al., 2024). Concurrently, the Elastic Net method effectively filtered high-dimensional proteomics data, with diagnostic AUC between 0.930 and 0.94 (Deivasigamani et al., 2025; Huang et al., 2025).

#### 3.2.2 Deep learning models

Deep learning algorithms like deep neural networks (DNNs) can learn complex nonlinear mappings when trained on large cohorts. Direct head-to-head comparisons revealed that a DNN model performed better than seven different traditional ML models in classifying CN vs. MCI, with an accuracy of 0.995 and an F1-score of 0.996 (Wang et al., 2025). For non-tabular data, specialized DL models are mandatory. For instance, a three-layer Feed-Forward Neural Network optimized to parse Surface-Enhanced Raman Scattering spectral data, achieving a striking accuracy of 99.5% in distinguishing AD from healthy controls (Kim et al., 2024).

#### 3.2.3 Multimodal fusion strategies

The integration of multimodal data is essential for comprehensive Alzheimer’s disease assessment, requiring effective machine learning fusion strategies. Early fusion with CatBoost is optimal for AD status prediction, and late fusion with LightGBM was found to be best for age-at-onset prediction (Ngai et al., 2024). A multimodal framework was developed to integrate MRI, PET, plasma biomarkers, and genetic data through CNNs, LSTMs, and GNNs. The model achieved an AUC of 0.94, surpassing unimodal performance and predicting 3-year conversion risk (Mohammed and Malhotra, 2025).

#### 3.2.4 Explainable AI methods

To overcome the “black-box” AI models, explainable AI (XAI) techniques are crucial for enhancing the transparency and clinical acceptability of blood-based biomarker models. SHapley Additive exPlanations (SHAP) are widely used to quantify feature contributions. In a large-scale study, SHAP analysis identified APOE ε4 as the most predictive feature, with an importance approximately 2.5 times greater than GFAP (Ngai et al., 2024). SHAP also validated that plasma p-tau217 is the most significant predictor of tau-PET load, outperforming clinical and MRI variables (Karlsson et al., 2025).

For complex ML models, the Integrated Gradients method has been applied to a SERS-enabled diagnostic platform, revealing critical metabolite peaks such as N-acetylneuraminic acid, which correlate with AD neuropathology (Kim et al., 2024). Lastly, simple visual frameworks, such as Classification and Regression tree (CART), have demonstrated that the plasma Aβ40/Aβ42 ratio alone was able to classify amyloid-PET status (Vergallo et al., 2019).

### 3.3 Optimized integration strategies: the step-care” framework

By balancing diagnostic performance, computational power, and financial costs identified across the literature, we map a unified, clinically actionable “Step-Care” framework across the AD continuum (Figure 2). This tiered workflow tailors diagnostic strategies to distinct clinical scenarios: cost-effective p-tau217 with computationally efficient models (e.g., logistic regression) to filter the population at risk in primary care; accurate diagnosis and staging at specialized centers with multi-biomarker panels in combination with ensemble and deep learning models; and multimodal fusion models for complex diagnoses and treatment decision-making.

**FIGURE 2:**
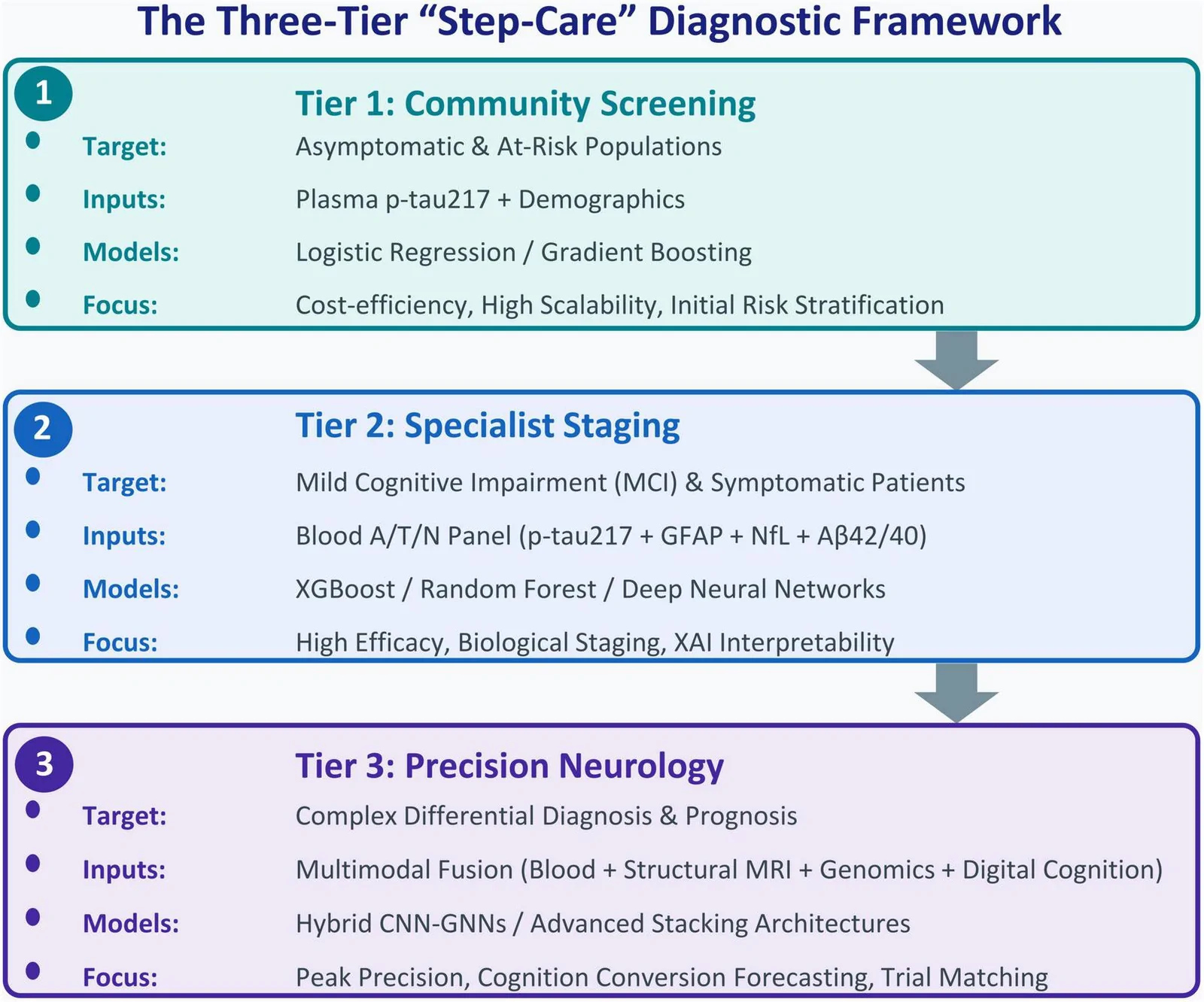
The three-tier “Step-Care” framework for Alzheimer’s disease. Tier 1 (Screening), Tier 2 (Diagnosis), and Tier 3 (Precision Staging). A/T/N, Amyloid, Tau, and Neurodegeneration; Aβ42/40, Amyloid-beta 42/40 Ratio; APOE ε4, Apolipoprotein E Epsilon 4; AUC, Area Under the Curve; AD, Alzheimer’s Disease; CNN, Convolutional Neural Network; CN, cognitively normal; DL, deep learning; GFAP, glial fibrillary acidic protein; GNN: graph neural network; LSTM, Long Short-Term Memory; MCI: mild cognitive impairment; ML, machine learning; MRI, magnetic resonance imaging; NfL, neurofilament light chain; PET, Positron Emission Tomography; p-tau181, Phosphorylated Tau at Threonine 181; p-tau217, Phosphorylated Tau at Threonine 217; SHAP, SHapley Additive exPlanations; SVM, Support Vector Machine; XAI, Explainable Artificial Intelligence; XGBoost, Extreme Gradient Boosting.

As illustrated in Figure 2, each tier of the framework is characterized by a specific target population, input features, analytical models, and focus. Tier 1 (Community Screening) targets asymptomatic and at-risk populations, using plasma p-tau217 and demographics as inputs, employing Logistic Regression or Gradient Boosting models, with a focus on cost-efficiency, high scalability, and initial risk stratification. Tier 2 (Specialist Staging) targets MCI and symptomatic patients, using a blood A/T/N panel as inputs, employing XGBoost, Random Forest, or Deep Neural Networks, with a focus on high efficacy, biological staging, and XAI interpretability. Tier 3 (Precision Neurology) targets complex differential diagnosis and prognosis cases, using multimodal fusion data as inputs, employing hybrid CNN-GNNs or advanced stacking architectures, with a focus on peak precision, cognition conversion forecasting, and trial matching.

## 4 Discussion

This paper reviews the integration of ML into blood biomarkers for diagnosis, prediction, and staging of Alzheimer’s disease. While plasma p-tau217 stands out as the most robust single diagnostic biomarker in predicting the burden of cerebral tau protein deposition and cognitive decline, the reliance on isolated biomarkers may overlook the multifactorial nature of AD pathology. Therefore, processing multimodal data via artificial intelligence helps capture the complex interactions among amyloidosis, tauopathy, neuroinflammation, and degeneration.

To translate these computational innovations into sustainable clinical workflows, we propose a hierarchical, clinically actionable “Step-Care” diagnostic framework, which offers a highly scalable solution that translates multimodal AI and blood-based biomarkers into routine, personalized clinical practice across the Alzheimer’s disease continuum. Furthermore, this framework holds profound implications for optimizing clinical trials. Before enrollment, ML models can pre-screen high-risk candidates to reduce screening failures. During trials, longitudinal tracking of surrogate blood markers such as GFAP and NfL via AI can quantitatively monitor therapeutic responses and disease progression.

Despite the promising applications, several critical barriers may impede the widespread clinical adoption of AI-biomarker models. First, variations in absolute levels of blood biomarkers depending on the specific platform used for testing, together with non-uniform approaches to creating machine learning models, their training, feature engineering, and validation, prevent researchers from replicating results obtained in different studies. Standardization should be addressed now more than ever. Additionally, many models currently being developed are trained on particular populations that may not be representative of a large portion of the population. The lack of generalizability emphasizes the urgent need for diverse, multi-ethnic training datasets. Furthermore, the inherent “black-box” nature of deep neural networks remains a critical barrier to clinical adoption. Future research should prioritize explainable frameworks that balance predictive accuracy with biological plausibility.

Specifically, to address standardization and reproducibility challenges, we propose several actionable recommendations. First, pre-analytical protocols should be harmonized, including standardized procedures for blood collection, processing times, centrifugation parameters, and storage conditions to reduce pre-analytical variations in blood biomarkers. Second, cross-platform calibration using certified reference materials and inter-laboratory proficiency testing would enhance comparison across studies. Third, establish standardized reporting guidelines for ML-based biomarker studies, defining minimum requirements for dataset description, feature engineering, model architecture, validation strategies, and performance metrics, which may enhance reproducibility. Fourth, adopt open-science practices, such as sharing de-identified datasets and model code in public repositories, to accelerate independent validation.

Regarding clinical implementation, several practical considerations should be addressed. First, regulatory pathways for AI-based *in vitro* diagnostic devices remain incompletely defined. Clear guidance on software as a medical device classification, validation requirements, and post-market surveillance would accelerate translation. Second, integration with existing clinical workflows is essential. Specifically, AI-biomarker tools should interface efficiently with electronic health record systems to minimize disruption. Third, cost-effectiveness analyses are required to justify the use of these approaches against standard diagnostic workups, particularly for Tier 1 screening applications. Fourth, clinician training programs are needed to help healthcare providers understand the capabilities and limitations of AI-assisted diagnostic tools. Finally, equity considerations must be prioritized to ensure these technologies are accessible and validated across diverse populations and prevent exacerbation of health disparities.

The contributions and implications of this review are as follows. Firstly, moving beyond conventional isolated assessments, on the basis of existing blood markers, we introduce new potential biomarkers and their combinations, highlighting their relevance for the early detection of AD. Secondly, by comparing existing AI- or machine-learning-based models for the diagnosis and staging of AD, this paper highlights the benefits of combining multiple models in this process. Crucially, we systematically pair biomarkers with their optimal computational architectures, thus providing more options for personalized diagnostics. Lastly, this paper enlarges the scope of available clinical information required to construct diagnostic and prognostic models for AD by introducing a clinically actionable “Step-Care” framework.

There are, however, certain limitations to this review. Firstly, due to the heterogeneity of clinical endpoints, diagnostic standards, and performance metrics across the literature, a meta-analysis could not be performed, preventing a quantitative synthesis of diagnostic accuracy. Second, the included literature relies primarily on cross-sectional data; this review offers limited insights into the long-term, longitudinal performance of these models for predicting real-world treatment outcomes over extended clinical timelines.

## 5 Conclusion

To conclude, blood biomarkers and machine learning models offer a useful toolkit for AD analysis. We propose a hierarchical diagnostic framework, transitioning from a cost-efficient single biomarker for preclinical screening to a sophisticated, multimodal system for precise diagnosis and staging. Optimal diagnostic performance requires a tailored selection of application scenarios, data availability, and computational robustness. The convergence of blood biomarkers, machine learning, and explainable AI represents a transformative paradigm in AD and provides a cornerstone for the next generation of precision neurology.
